# BRCA1 alleviates inflammation, oxidative stress, and ovarian granulosa cell apoptosis by inhibiting endoplasmic reticulum stress, thereby ameliorating polycystic ovary syndrome

**DOI:** 10.1186/s40001-025-03734-6

**Published:** 2026-01-03

**Authors:** Xiaolan Huang, Shuyin Zhang, Danhong Liang, Lingling Qiu, Ruiyun Wu, Rong Wei, Xiaoqing Chen, Suzhen Huang

**Affiliations:** 1 Department of Reproductive Medicine, The Second Affiliated Hospital of Fujian Medical University, 34 North ZhongshanRoad, Licheng District, Quanzhou, Fujian China; 2 Department of Rheumatology and Immunology, The Second Affiliated Hospital of Fujian Medical University, 34 North ZhongshanRoad, Licheng District, Quanzhou, Fujian China

**Keywords:** Polycystic ovary syndrome, BRCA1, Endoplasmic reticulum stress, Oxidative stress, Inflammation

## Abstract

Polycystic ovary syndrome (PCOS) is a prevalent endocrine disorder characterized by metabolic imbalance, oxidative stress, and granulosa cell (GC) dysfunction. Given the established role of BRCA1 in maintaining genomic stability and regulating stress responses, its potential involvement in PCOS pathogenesis warrants investigation. We examined the impact of BRCA1 on PCOS using a dehydroepiandrosterone (DHEA)-induced mouse model and testosterone-stimulated KGN cells. Lentiviral vectors overexpressing BRCA1 were administered by ovarian injection in PCOS mice or used to infect KGN cells. The IL-1β, TNF-α, and IL-6 levels were measured using enzyme-linked immunosorbent assay. Western blotting was performed to evaluate the expression levels of BRCA1 and other proteins related to inflammation, apoptosis, and endoplasmic reticulum stress (ERS). A TUNEL assay and flow cytometry were used to assess cell apoptosis. Spectrophotometry was used to measure the levels of reactive oxygen species, malondialdehyde, superoxide dismutase, and catalase. Cell survival was evaluated using a CCK-8 assay. Hematoxylin and eosin staining was used to assess the pathological alterations in the ovaries of PCOS mice. Additionally, rescue experiments were conducted on KGN cells treated with the ERS inducer thapsigargin (TG) to determine whether the protective effects of BRCA1 overexpression could be reversed through reactivating ERS. BRCA1 expression was reduced in PCOS. BRCA1 overexpression normalized sex hormone levels, improved ovarian morphology, and attenuated inflammatory signaling, oxidative stress, apoptosis, and ERS in vivo and in vitro. Notably, the ERS inducer TG reversed these protective effects, indicating ERS dependence. These findings suggest that BRCA1 mitigates PCOS phenotypes primarily by suppressing ERS and downstream inflammatory/oxidative and apoptotic pathways, thereby highlighting BRCA1 as a potential molecular target for PCOS therapy.

**Supplementary Information:**

The online version contains supplementary material available at 10.1186/s40001-025-03734-6.

## Introduction

Polycystic ovary syndrome (PCOS) affects 10–15% of women during their reproductive years and is one of the leading causes of infertility [1]. PCOS is often associated with obesity and insulin resistance, which increase the risk of type 2 diabetes, cardiovascular disease, and endometrial cancer [2]. The exact causes of PCOS remain unclear, involving genetic, epigenetic, and environmental factors [3]. Consequently, no definitive treatments are available for PCOS, and precise therapies targeting the underlying causes are lacking.

PCOS is associated with chronic inflammation and oxidative stress. Ovarian inflammation and reactive oxygen species (ROS) levels are substantially higher in patients with PCOS than in healthy individuals [4–6]. The serum levels of proinflammatory cytokines such as C-reactive protein, interleukin-6 (IL-6), IL-18, and tumor necrosis factor-α (TNF-α) are elevated, whereas the levels of anti-inflammatory cytokines such as IL-10 and IL-27 are reduced [7]. The extensive inflammatory cell infiltration in the ovaries of PCOS patients suggests a link with abnormal follicle development [8]. Increased serum malondialdehyde (MDA) and ROS levels, along with decreased total antioxidant capacity in those with PCOS, further indicate a role of ROS in abnormal follicular development [9]. Hence, ovarian oxidative stress and inflammation must be controlled to improve oocyte development in patients with PCOS.

Granulosa cells (GCs) surround oocytes during the primordial follicle stage and provide essential nutrients for oocyte development [10]. Reduced numbers of GCs, impaired GC function, or disrupted connections of GCs with oocytes can reduce oocyte quality, impair ovarian reserve, and lead to the development of PCOS [11]. Inflammation in the follicular fluid impairs GC function and affects oocyte development [12]. Increased oxidative stress promotes GC apoptosis [13], reducing the ability of GCs to resist ROS and leading to fewer, poorer-quality follicles as well as abnormal ovarian endocrine function [14]. Reducing inflammation and oxidative stress in GCs to strengthen GC function and increase oocyte quality is the key to treating PCOS.

BRCA1 is a tumor-suppressor gene located on chromosome 17 that plays crucial roles in cell replication and growth [15]. BRCA1 mutations lead to various cancers, such as breast and ovarian cancers [16]. BRCA1 maintains genomic stability by repairing double-strand breaks via homologous recombination [17]. A recent case–control study reported an association between BRCA1 variants and PCOS in reproductive-aged women [18]. Moreover, recent research has shown that BRCA1 regulates oxidative stress; its mutation or deletion increases ROS production, whereas BRCA1 overexpression inhibits ROS production [19, 20]. Additionally, reduced BRCA1 expression levels promote inflammatory responses [21]. These findings suggest that BRCA1 influences the progression of PCOS through regulating oxidative stress and inflammation; however, the mechanisms underlying these effects require further study.

In this study, we investigated the role of BRCA1 in thePCOS and explored the mechanisms underlying its effects. Mouse and cellular models of PCOS were established to evaluate BRCA1 expression and its regulatory impact on inflammation and oxidative stress. The expression of endoplasmic reticulum stress (ERS)-related proteins in the ovaries of PCOS mice and KGN cells was analyzed to clarify the potential involvement of ERS. Furthermore, rescue experiments using the ERS inducer thapsigargin (TG) were performed to verify whether BRCA1 ameliorates PCOS by suppressing ERS, thereby reducing inflammation, oxidative stress, and apoptosis. Collectively, this study provides new molecular insights into the protective role of BRCA1 and suggests its potential as a therapeutic target for the prevention and management of PCOS.

## Methods

### Establishing PCOS mouse model

Four-week-old female C57BL/6 J mice were obtained from Slac Laboratory Animal Co., Ltd. (Shanghai, China). The mice were randomly divided into control (*n* = 10) and PCOS (*n* = 20) groups. The PCOS group of mice received a daily injection of 0.1 mL dehydroepiandrosterone (DHEA) dissolved in sesame oil at a dosage of 6 mg/kg for a continuous period of 21 days to establish the PCOS mouse model as previously described [22]. The control group mice received a subcutaneous injection of sesame oil in a volume equal to that of the injection received by the PCOS mice. The criteria for mice with PCOS are elevated androgen levels, irregular ovulation patterns, and the presence of polycystic alterations in their ovaries.

### Animal grouping and treatment

The PCOS mice were randomly allocated to PCOS and PCOS + Lv-BRCA1 groups, each consisting of 10 mice. A control group was included in the subsequent experiments. Lentiviral vectors overexpressing BRCA1 (Lv-BRCA1) and control lentiviral vectors (Lv-NC) were purchased from GeneChem (Shanghai, China). Mice in the PCOS + Lv-BRCA1 group received a 10 µL subovarian capsule injection of the appropriate lentivirus, with a titer of 1 × 10^8^ TU/mL. The muscle and skin tissues were sutured layer-by-layer after the injection. Two weeks after injection, the mice were fasted for 12 h, blood was collected from the orbit, the mice were euthanized, and the ovarian tissues were harvested.

### Cells and treatments

The human ovarian granulosa cell line KGN was purchased from Pricella Biotechnology Co., Ltd. (Wuhan, China) and was cultured in DMEM/F12 (iCell, Shanghai, China) containing 10% fetal bovine serum (Yuanye, Shanghai, China) and 1% penicillin–streptomycin (Yeasen, Shanghai, China) at 37 °C. The cellular model of PCOS was established by treating KGN cells with 10 µM testosterone (Acmec, Shanghai, China) for one day, as previously described [23]. This treatment mimics the hyperandrogenic environment of PCOS and reproduces key pathological features in GCs. To clarify the mechanism of BRCA1 on the apoptosis, oxidative stress, and inflammation of GCs, the ERS inducer TG (500 nM) (Macklin, Shanghai, China) was introduced. All in vitro experiments using KGN cells were independently repeated three times (biological replicates).

### H&E staining

The ovarian tissues were fixed in formalin and sliced into 4-µm sections. The paraffin-embedded sections were dewaxed and rehydrated. Sequential staining was performed using H&E (Biolab, Wuhan, China). The sections were air-dried and mounted with neutral gum (Meryer, Shanghai, China). The morphology of the ovaries in each group was observed under a microscope (Nikon Eclipse Ci-E, Tokyo, Japan) by counting the number of normal and cystic follicles as well as corpora lutea in randomly selected sections of each ovary.

### Serum hormone level measurement

A biochemical analyzer (IDEXX, ME, USA) was used to measure the levels of luteinizing hormone (LH), testosterone (T), follicle-stimulating hormone (FSH), anti-Müllerian hormone (AMH), and estradiol (E2) in the serum of the mice in each group.

### Immunofluorescence staining

Deparaffinized and rehydrated paraffin sections of mouse ovarian tissue were immersed in citrate buffer (Attobio, Suzhou, China) and heated in a microwave oven for retrieving the antigens. The sections were naturally cooled and placed in a wet box. Then, 10% bovine serum albumin (Sigma Aldrich, St. Louis, MO, USA) solution was added, and the sections were blocked at 37 °C for 30 min. The sections were rinsed, and the sections were sequentially incubated with primary antibody anti-BRCA1 (1:300, bs-0803R, Bioss, Beijing, China) and secondary antibody goat anti-rabbit IgG H&L (FITC; 1:2000, ab6717, Abcam, Cambridge, UK). The sections were mounted with a medium containing DAPI (Biosharp, Hefei, China) and observed under a microscope (Olympus, Tokyo, Japan).

### Western blotting

The total protein was extracted from the ovarian tissues or KGN cells, and the bicinchoninic acid method was used to determine the protein concentration. The protein concentration in the samples was adjusted to the same concentration. The samples were subjected to polyacrylamide gel electrophoresis and transferred onto a PVDF membrane (Millipore, Billerica, MA, USA). The membranes were blocked with 1% BSA. The PVDF membranes were washed with TBST and then exposed to diluted primary antibodies and incubated overnight at 4 °C. The membranes were incubated with secondary antibodies the following day. Finally, ECL solution (Beyotime, Beijing, China) was added to the membranes, and the protein bands were observed and captured using a chemiluminescence gel imager (Tanon 2500, Shanghai, China). The protein bands were analyzed using ImageJ software. The antibodies used in this study are listed in Table 1.

**Table 1:** Information of antibodies used in Western blotting

Name	Manufacturer	Product code	Dilution rate
BRCA1	Novus	NBP1-41185	1:2000
iNOS	Cell signaling technology	#2982	1:1000
Cox2	Cell signaling technology	#4842	1:1000
Bax	Cell signaling technology	#2772	1:1000
Bcl-2	Cell signaling technology	#3498	1:1000
eIF2α	Cell signaling technology	#9722	1:1000
p-eIF2α	Cell signaling technology	#9721	1:1000
ATF4	Cell signaling technology	#11815	1:1000
CHOP	Cell signaling technology	#5554	1:1000
β-actin	Abcam	ab8226	1:10000
Goat anti-rabbit IgG	Abcam	ab6721	1:10000
Goat anti-mouse IgG	Abcam	ab205719	1:10000

### Enzyme linked immunosorbent assay (ELISA)

The concentrations of IL-1β, TNF-α, and IL-6 were measured in the serum and ovarian tissues of mice, as well as the culture supernatant of KGN cells, following the guidelines provided with the ELISA kit (Beyotime, Beijing, China).

### TUNEL staining

TUNEL assay was conducted with a TUNEL Apoptosis Detection Kit (Absin, Shanghai, China) according to the instructions provided by the manufacturer. Briefly, the ovarian tissue sections were dewaxed, rehydrated, and placed in an antigen retrieval solution. The sections were cooled and then washed with phosphate-buffered saline and treated with proteinase K and 0.3% Triton X-100 at 37 °C for 20 min. TUNEL detection solution was added and reacted with the sections at 37 °C in the dark for 1 h. Mounting medium was applied, and the sections were examined under a fluorescence microscope (Nikon Eclipse Ci-E, Tokyo, Japan).

### ROS detection

The ROS levels in the mouse ovarian tissues and KGN cells were assessed using an ROS ELISA kit (Giled, Wuhan, China). The ovarian tissues and KGN cells were processed into homogenates and cell lysates, respectively, and handled according to the kit instructions. The OD_450nm_ of the standards and samples was measured using spectrophotometry, and the concentrations of ROS in the ovarian tissues and KGN cells were calculated based on a standard curve.

### Detecting markers of oxidative stress

The mouse ovarian tissues were homogenized, and KGN cells were sonicated. The levels of the markers of oxidative stress were determined using spectrophotometry according to the instructions provided with the MDA, superoxide dismutase (SOD), and catalase (CAT) detection kits (Jiancheng, Nanjing, China).

### CCK-8 assay

KGN cells were seeded in 96-well plates at a density of 5 × 10 cells/well and treated according to the experimental grouping, with five replicate wells per group. The medium was refreshed after 12, 24, and 48 h, and 10 µL CCK-8 (Absin, Shanghai, China) was introduced. The cells were then incubated for an additional 2 h. The OD_450_ was measured using a microplate reader (Tecan Spark, Männedorf, Switzerland).

### Flow cytometry

The KGN cells were cultured in a 6-well plate at a seeding density of 1 × 10^5^ cells/well and treated according to the experimental grouping after adherence. The cells were then harvested using precooled phosphate-buffered saline in a flow tube and centrifuged at 1000 × *g* for 5 min. Then, 1 × 10^6^ cells were collected and centrifuged again, and the cell pellet was collected. Then, 195 μL of the binding solution and 5 μL of Annexin V-FITC and PI solution (Beyotime, Beijing, China) were added to incubate the cells in the dark for 30 min. Three replicates were used for each group. In addition, three single stains of Annexin V-FITC and PI as negative controls and three blank controls were used. A flow cytometer (Beckman Coulter, Brea, CA, USA) was used for observation and analysis.

### Statistical analysis

Statistical analyses were performed using SPSS version 23.0. All quantitative data are presented as mean ± standard deviation. Differences between groups were analyzed using the independent-samples *t*-test and among groups using one-way ANOVA with Tukey’s post hoc test. *P* < 0.05 was considered statistically significant. A formal power analysis was not performed; however, the sample sizes were consistent with those of previous studies and adequate to ensure statistical reliability.

## Results

### BRCA1 expression is reduced in PCOS

**Fig. 1:**
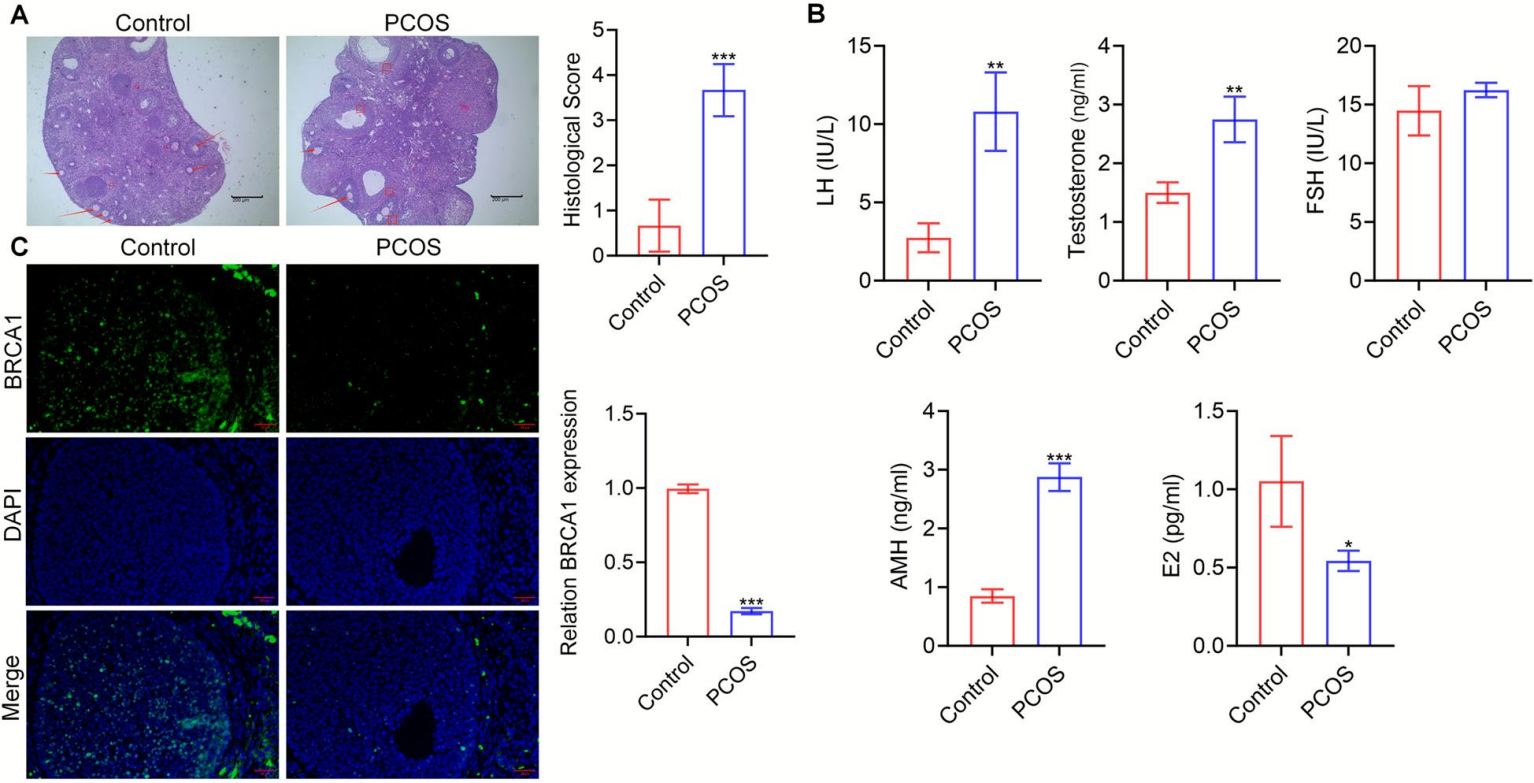
The level of BRCA1 is decreased in PCOS. **A** Representative H&E-stained ovarian sections from Control and PCOS mice. Arrows indicate normal follicles; CL means corpora lutea; indicates cystic follicles. Scale bar = 200 μm. **B** The contents of LH, T, FSH, AMH, and E2 were tested to verify the successful construction of the PCOS model. * P < 0.05, ** *P* < 0.01, *** P < 0.001. **C** The expression level of BRCA1 in ovarian tissues of mice in Control and PCOS groups was determined by immunofluorescent staining. Scale bar = 20 μm. Data are expressed as mean ± SD (*n* = 10 per group)

We developed a mouse model to determine the involvement of BRCA1 in PCOS. H&E staining revealed morphological differences between the control and PCOS groups. The follicular architecture of the control ovaries was normal, with follicles at multiple developmental stages and visible corpora lutea, consistent with ovulation. In contrast, the PCOS ovaries contained numerous cystic follicles with thinned granulosa cell layers and an absence of corpora lutea, indicative of anovulation and polycystic morphology (Fig. 1A). In addition, the serum levels of LH, T, and AMH were markedly higher, and the estradiol level was lower in PCOS mice than in the control. Although the level of FSH was higher in PCOS mice, the difference was not significant (*P* > 0.05; Fig. 1B). These results demonstrated that a mouse model of PCOS was successfully constructed. Furthermore, the BRCA1 expression level in the ovaries of PCOS mice was lower than that in control mice (Fig. 1C).

### BRCA1 alleviated inflammatory response in PCOS mice

**Fig. 2:**
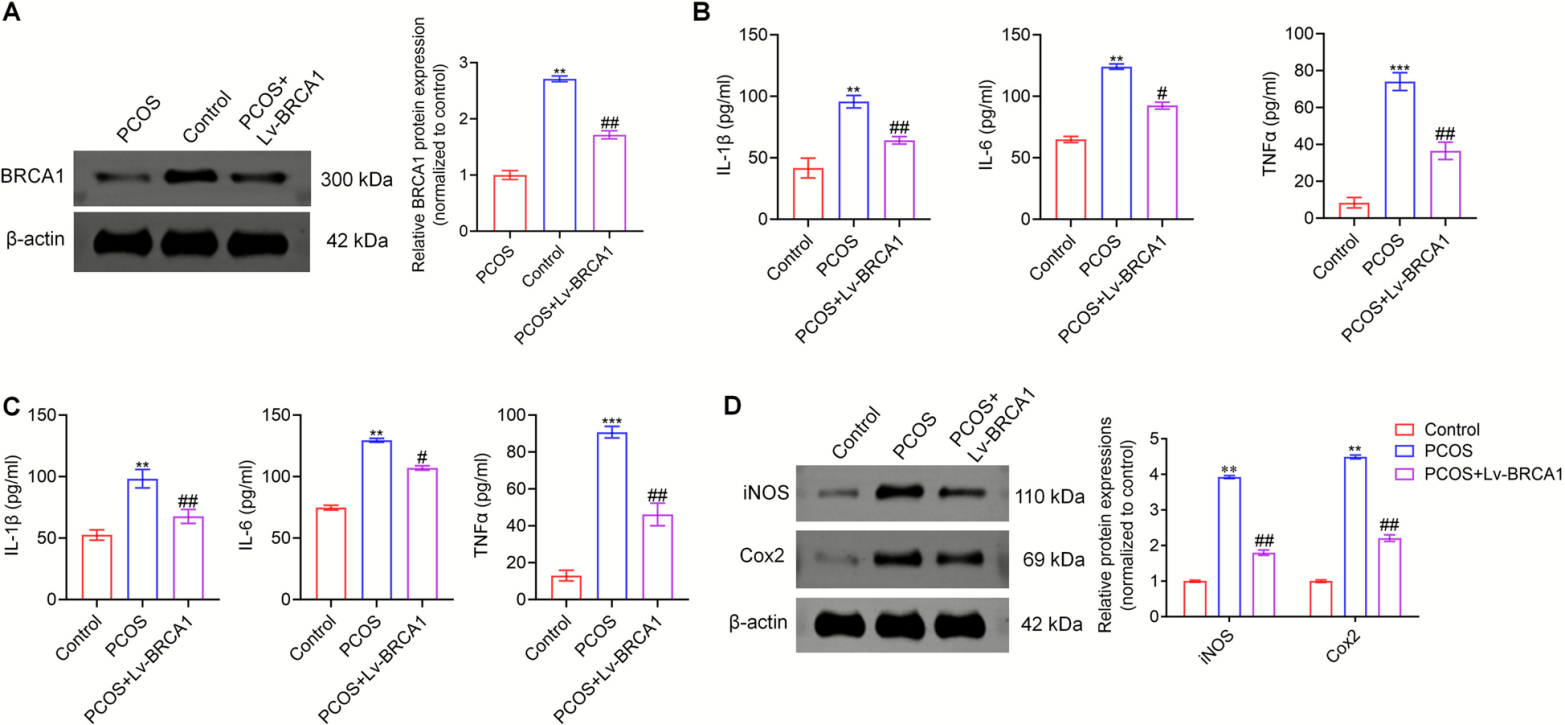
BRCA1 alleviates the inflammatory response in PCOS mice. **A** The overexpression efficiency of Lv-BRCA1 was examined by Western blot. The levels of IL-1β, TNF-α, and IL-6 in the serum (**B**) and ovaries of PCOS mice (**C**) were determined by ELISA.** D** The expression level of inflammation-related proteins iNOS and Cox2 was evaluated by Western blot. ** *P* < 0.01, *** P < 0.001 *vs* Control group, #* P* < 0.05, ## *P* < 0.01 *vs* PCOS group. Data are expressed as mean ± SD (*n* = 10 per group)

We investigated the role of BRCA1 in PCOS by overexpressing BRCA1 in PCOS mice using a lentivirus. Western blot analysis confirmed that injecting Lv-BRCA1 substantially increased the BRCA1 level in the ovaries of the PCOS mice (Fig. 2A). Furthermore, BRCA1 overexpression reduced the levels of IL-1β, TNF-α, and IL-6 in the serum and ovaries of mice with PCOS (Fig. 2B). In addition, BRCA1 overexpression reduced the expression level of the inflammation-related proteins iNOS and Cox2 in the ovaries of PCOS mice compared with the controls (Fig. 2C). These findings suggest that BRCA1 alleviates inflammation in mice with PCOS.

### BRCA1 alleviated the PCOS symptoms in mice and reduced cell apoptosis in ovaries

**Fig. 3:**
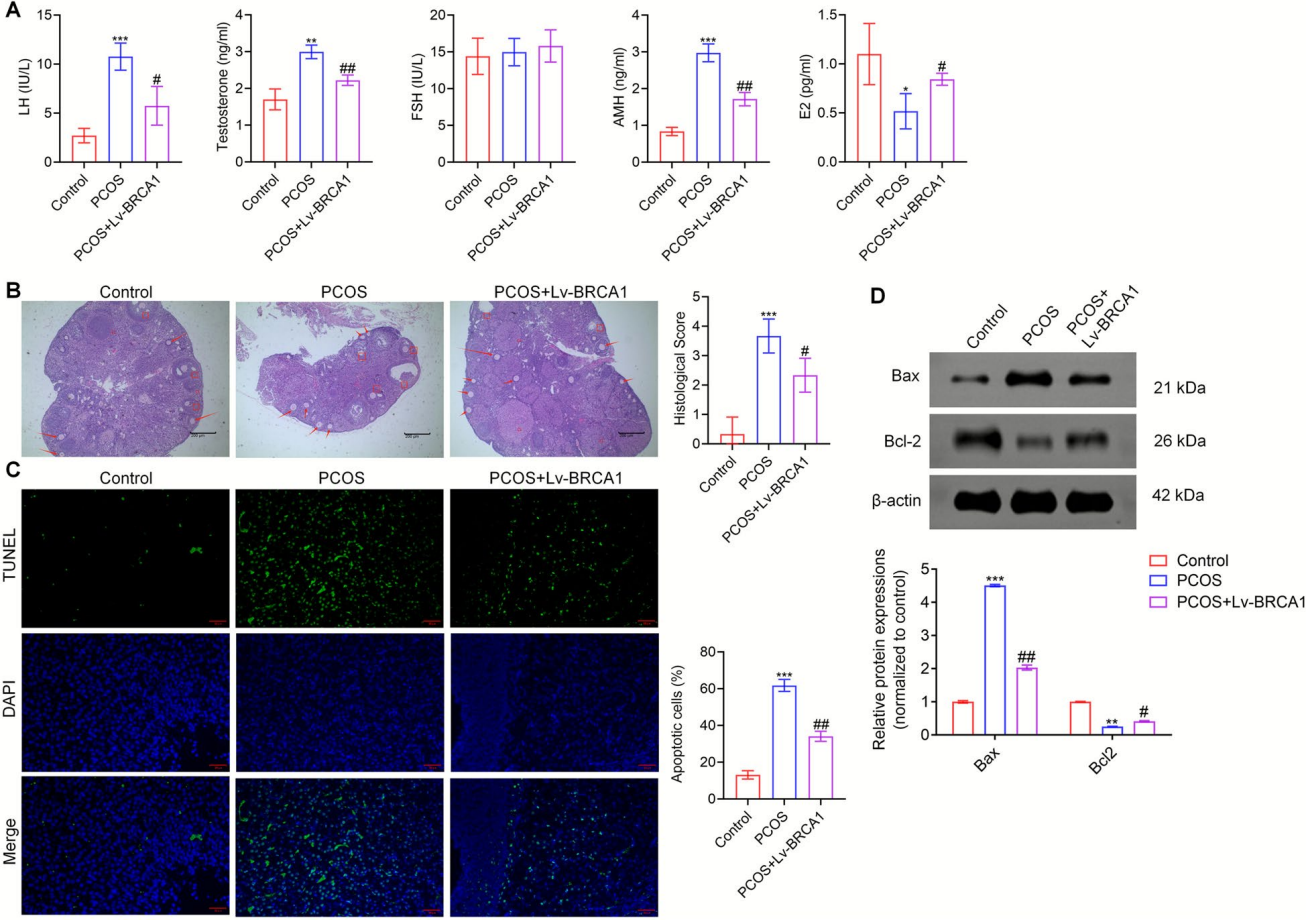
BRCA1 alleviates the symptoms of PCOS mice and reduces apoptosis in the ovaries. **A** The levels of sex hormones in the serum of mice in Control, PCOS, and PCOS + Lv-BRCA1 groups were tested. **B** Representative H&E-stained ovarian sections from Control, PCOS, and PCOS + Lv-BRCA1 mice. Arrows indicate normal follicles; CL means corpora lutea; indicates cystic follicles. Scale bar = 200 μm. **C** TUNEL staining was performed to evaluate the apoptosis in the ovaries. Scale bar = 50 μm. **D** The expressions of apoptosis-related proteins Bax and Bcl-2 were detected by Western blot. * P < 0.05, ** *P* < 0.01, *** P < 0.001 *vs* Control group, #* P* < 0.05, ## *P* < 0.01 *vs* PCOS group. Data are expressed as mean ± SD (*n* = 10 per group)

We measured the levels of sex hormones in the mouse serum. The serum levels of luteinizing hormone, testosterone, and AMH were lower, and the estradiol levels were higher in BRCA1-overexpressing PCOS mice than in the PCOS group. However, the follicle-stimulating hormone levels did not differ between the groups (Fig. 3A). Phenotypic assessment (Fig. S1) revealed that BRCA1 overexpression partially reversed the PCOS-associated increases in body weight, prolonged diestrus, and metabolic disturbances. H&E staining showed that the ovarian lesions in the PCOS + Lv-BRCA1 group were markedly reduced compared with those in the PCOS group. The ovaries of the PCOS mice exhibited numerous cystic follicles and a loss of corpora lutea, whereas BRCA1 overexpression partially restored the normal follicular morphology and the presence of corpora lutea in these mice, indicating an improvement in ovarian structure (Fig. 3B).

BRCA1 overexpression also inhibited apoptosis. The number of TUNEL-positive cells in the PCOS + Lv-BRCA1 group was lower than in the PCOS group (Fig. 3C). Additionally, the expression levels of Bax and Bcl-2 were lower and higher, respectively, in the PCOS + Lv-BRCA1 group of mice than in the PCOS group (Fig. 3D). These results suggest that BRCA1 alleviates the aberrant levels of sex hormones, reduces the severity of ovarian lesions, and decreases apoptosis in the ovaries in PCOS mice.

### BRCA1 reduced levels of oxidative and ERS in ovaries of PCOS mice

**Fig. 4:**
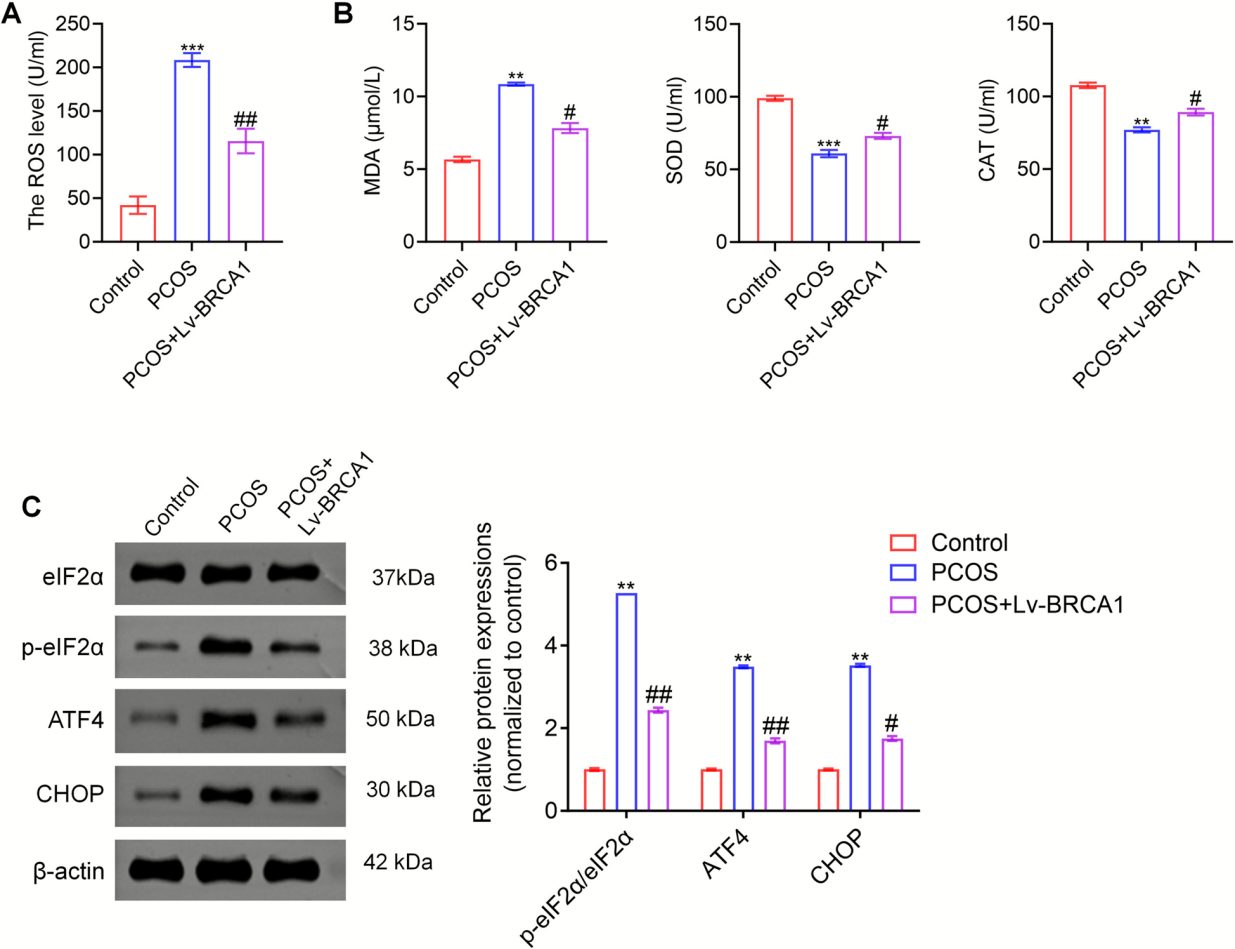
BRCA1 reduces OS levels and ERS in ovaries of PCOS mice. **A** The ROS level in the ovaries of these three groups of mice was determined by ELISA. **B** The contents of MDA, SOD, and CAT were detected using corresponding kits.** C** The expression levels of eIF2α, p-eIF2α, ATF4, and CHOP were evaluated by Western blot. ** *P* < 0.01, *** P < 0.001 *vs* Control group, #* P* < 0.05, ## *P* < 0.01 *vs* PCOS group. Data are expressed as mean ± SD (*n* = 10 per group)

We further examined the molecular mechanism through which BRCA1 alleviates the symptoms of PCOS by measuring the level of oxidative stress and the occurrence of ERS in the ovaries of mice before and after BRCA1 overexpression. The ROS content in the ovaries of PCOS mice was substantially higher than that in the ovaries of healthy mice, whereas BRCA1 overexpression resulted in a notable decrease in ROS content compared with that in control mice (Fig. 4A). Additionally, the MDA content was higher and the SOD and CAT levels were lower in the ovaries of PCOS mice compared with those in the control group. BRCA1 overexpression reduced the MDA levels and increased the SOD and CAT levels in these mice compared with those in the controls (Fig. 4B). Moreover, the expression levels of proteins associated with ERS were determined. The differences in the expression level of eIF2α were minimal between the ovaries of PCOS and control mice. However, the expression levels of p-eIF2α, ATF4, and CHOP were higher in the PCOS than in the control mice. No notable change was observed in the eIF2α expression level following BRCA1 overexpression in PCOS mice; however, the expression levels of p-eIF2α, ATF4, and CHOP remarkably decreased (Fig. 4C). These results indicate that BRCA1 reduced the oxidative and ERS levels in the ovaries of PCOS mice.

### BRCA1 inhibited apoptosis, inflammation, oxidative stress and ERS in KGN cell model

**Fig. 5:**
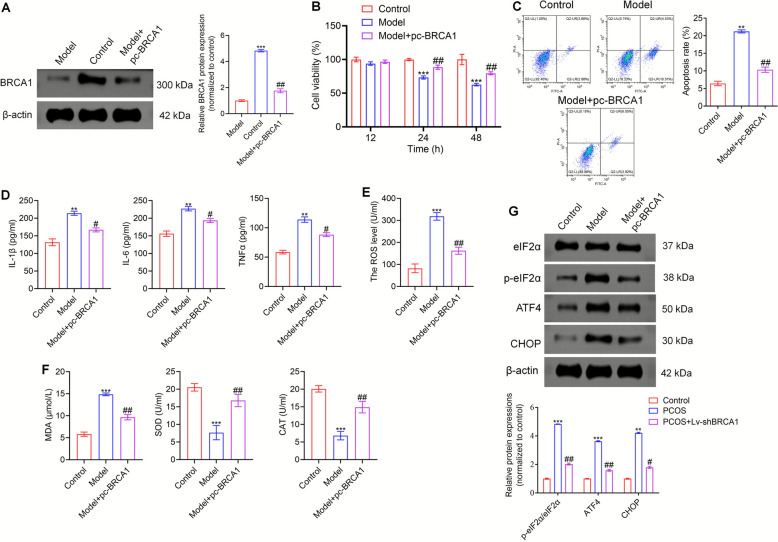
BRCA1 inhibits apoptosis, inflammation, oxidative stress, and ERS in KGN cell model. **A** Western blot was performed to verify the efficiency of pc-BRCA1. **B** Cell viability of KGN cells in Control, Model, and Model + pc-BRCA1 groups was assessed by CCK-8 assay. **C** Flow cytometry was conducted to detect the apoptosis level of KGN cells in these three groups. **D** The levels of IL-1β, TNF-α, and IL-6 secreted by KGN cells were determined by ELISA. **E** The ROS level in KGN cells was tested by ELISA. **F** The contents of MDA, SOD, and CAT were detected using corresponding kits. **G** The expression levels of eIF2α, p-eIF2α, ATF4, and CHOP were evaluated by Western blot. ** *P* < 0.01, *** P < 0.001 *vs* Control group, #* P* < 0.05, ## *P* < 0.01 *vs* Model group. Data represent three independent experiments (*n* = 3)

We examined the effects of BRCA1 in vitro. We created an in vitro PCOS cell model using testosterone to stimulate the KGN cells and overexpress BRCA1 in the KGN model. We confirmed BRCA1 was overexpressed in the KGN cell model using Western blotting (Fig. 5A). The cell viability was substantially higher (Fig. 5B) and apoptosis was inhibited (Fig. 5C) in the BRCA1-overexpressing KGN cell model. The testosterone-stimulated KGN cells secreted more of the inflammatory factors IL-1β, TNF-α, and IL-6, and BRCA1 overexpression inhibited the secretion of inflammatory factors of the KGN cells (Fig. 5D). In addition, BRCA1 overexpression reduced the level of ROS in the KGN cell model (Fig. 5E), decreased the MDA content, and enhanced the SOD and CAT levels (Fig. 5F). Moreover, the pattern of the protein levels associated with ERS in the KGN cells was similar to that observed in the *in viv*o studies. The eIF2α level was almost the same among the groups of KGN cells. The expression levels of p-eIF2α, ATF4, and CHOP were higher in the model group, which decreased after BRCA1 overexpression (Fig. 5G). These results indicated that BRCA1 inhibited apoptosis, inflammation, and oxidative stress as well as ERS in a testosterone-induced KGN cell model.

### BRCA1 alleviated cell apoptosis, inflammation and oxidative stress in GCs via inhibiting ERS

**Fig. 6:**
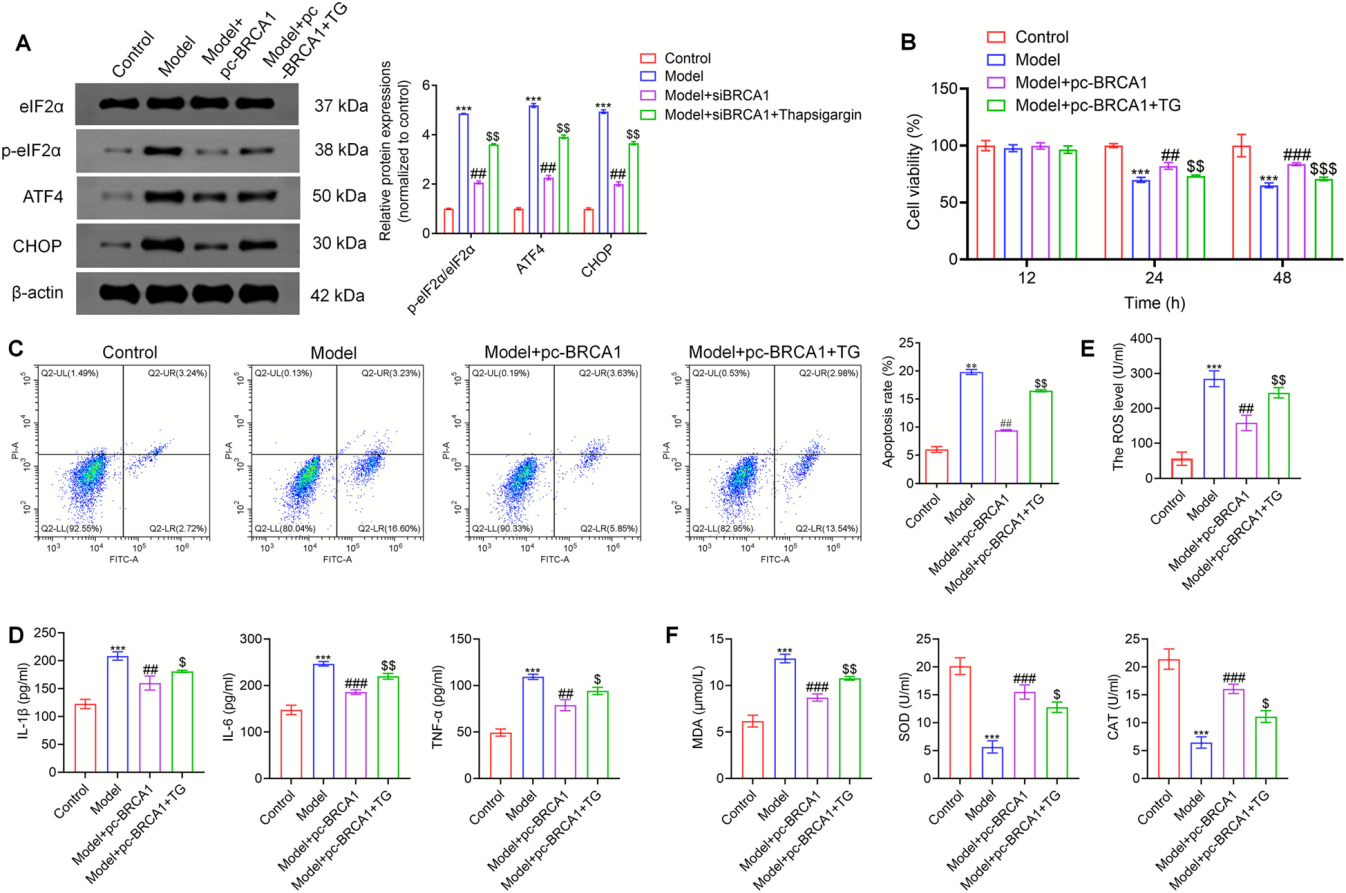
BRCA1 alleviates apoptosis, inflammation, and oxidative stress in GCs by inhibiting ERS. **A** The expression levels of ERS markers of KGN cells in Control, Model, Model + pc-BRCA1, and Model + pc-BRCA1 + TG groups were verified by Western blot. **B** Cell viability of KGN cells in these four groups was assessed by CCK-8 assay. **C** Flow cytometry was performed to detect the apoptosis level of KGN cells. **D** The levels of IL-1β, TNF-α, and IL-6 secreted by KGN cells were determined by ELISA. **E** The ROS level in KGN cells was tested by ELISA. **F** The contents of MDA, SOD, and CAT were detected using corresponding kits. ** *P* < 0.01, *** P < 0.001 *vs* Control group, ## *P* < 0.01, ###* P* < 0.001 *vs* Model group, $ *P* < 0.05, $$ *P* < 0.01, $$$* P < 0.001 *vs* Model + pc-BRCA1 group. Data represent three independent experiments (*n* = 3)

We treated KGN cells with the ERS inducer, TG, in a series of rescue experiments to gain a deeper understanding of the molecular mechanism underlying the action of BRCA1 and determine whether BRCA1 alleviates PCOS via inhibiting ERS. The KGN cells were divided into four groups: control, model, model + pc-BRCA1, and model + pc-BRCA1 + TG. The expression levels of ERS-related proteins were evaluated in each group of KGN cells. The expression levels of p-eIF2α, ATF4, and CHOP increased after TG treatment (Fig. 6A). The KGN cell vitality was lowest in the model group, whereas cell vitality was markedly higher in the BRCA1 overexpression group. However, cell viability decreased when KGN cells were treated with TG (Fig. 6B). The apoptosis experiment showed that the inhibitory effect of BRCA1 overexpression on KGN cell apoptosis was reversed by TG treatment (Fig. 6C). Additionally, higher levels of inflammatory factors secreted by KGN increased after TG treatment (Fig. 6D). The inhibitory action of BRCA1 overexpression on the oxidative stress of the KGN cells was reversed by TG treatment (Fig. 6E, F). These results suggest that BRCA1 alleviates cell apoptosis, inflammation, and oxidative stress in GCs by inhibiting ERS.

## Discussion

Most studies on BRCA1 have focused on the impact of BRCA1 mutations or deletions in various cancers; few studies have considered the relationship between BRCA1 and PCOS. Jiao et al. identified multiple mutations in BRCA1 in the ovaries of patients with PCOS and found that the genetic and epigenetic characteristics of the ovaries in patients with PCOS are similar to those of ovarian cancer [24]. Pujar et al. reported a strong association between BRCA1, but not BRCA2, variants and PCOS in reproductive-aged women [18]. We hypothesized that the regulation of PCOS involves BRCA1. In this study, we generated a mouse model of PCOS by administering DHEA and observed a reduction in the expression level of BRCA1 in the ovaries of PCOS mice. Interestingly, the mRNA and protein expression levels of BRCA1 have also been found to be reduced in sporadic ovarian and breast cancers [25, 26].

Whether the expression level of BRCA1 affects PCOS progression remains unknown. Here, we found that BRCA1 overexpression in PCOS mice improved clinical indicators, restoring the aberrant hormone levels in the serum. BRCA1 overexpression improved the polycystic morphology of the ovaries of PCOS mice, increased the number of corpora lutea, and substantially reduced the number of cystic follicles. Considering the association between chronic low-grade inflammation and OS and the development of abnormal follicles in PCOS, which also leads to increased apoptosis of GCs [27, 28], coupled with the vital role of GCs in oocyte development, it is inevitable that the increase in GC apoptosis is related to aberrant follicle development in PCOS [29]. Therefore, we constructed an in vitro PCOS cell model, in addition to the PCOS mouse model, to explore the effects of BRCA1 on GCs. We found that BRCA1 overexpression inhibited inflammation, oxidative stress, and apoptosis in PCOS mice and a KGN cell model. BRCA1 exerts anti-oxidative-stress and anti-inflammatory effects. BRCA1 deficiency promotes the activation of the NLRP3 inflammasome and thus breast cancer metastasis [21]. Promoting the BRCA1–NRF2 signaling pathway in hepatocellular carcinoma can reduce the ROS levels in cancer cells, protecting liver cancer cells from oxidative damage [20]. BRCA1 overexpression in hypoxia-induced neural stem cells reduced oxidative stress as well as apoptosis and stimulated the multiplication of neural stem cells [30]. Our study confirms that BRCA1 inhibits inflammation, oxidative stress, and apoptosis in ovaries and GCs in PCOS.

We also explored how BRCA1 inhibits inflammation, oxidative stress, and apoptosis in PCOS. ERS levels increase in the GCs of patients with PCOS [31, 32]. We observed that the expression levels of ERS-related proteins p-eIF2α, ATF4, and CHOP decreased when BRCA1 was overexpressed in the ovaries of PCOS mice and in the KGN cell model. Misfolded or unfolded proteins accumulate in the endoplasmic reticulum under pathological conditions, leading to stress. Three major signaling pathways of PERK, ATF6, and IRE1α are thus activated to mediate the unfolded protein response to alleviate ERS [33]. PERK is a transmembrane protein located in the endoplasmic reticulum. PERK is inactivated under normal conditions through binding to GRP78. However, PERK is released and phosphorylated under ERS [34]. Activated PERK initiates the transcription of a series of genes to alleviate ERS or initiates the cell apoptosis program through the eIF2α–ATF4–CHOP pathway [35]. BRCA1 mediates the PERK and IRE1 ubiquitination as E3 ubiquitin ligases. BRCA1 mutations or deletions increase PERK and IRE1 levels, activating the unfolded protein response [36]. In addition, BRCA1 overexpression inhibits GRP78 expression, whereas mutant BRCA1 or BRCA1 knockdown enhances GRP78 expression levels and promotes the unfolded protein response [37]. BRCA1 can reduce the ERS that occurs in PCOS through the PERK signaling pathway.

Persistent ERS increases the oxidative stress in cells and activates inflammatory responses, leading to cell apoptosis [38]. Hyperandrogenism induces ERS-mediated ferroptosis in GCs, contributing to ovarian dysfunction in PCOS [23]. We verified whether BRCA1 affects PCOS via regulating ERS by treating BRCA1-overexpressing KGN cells with the ERS inducer TG. Administering TG aggravated inflammation, oxidative stress, and apoptosis in the KGN cells. ERS plays a vital role in the pathogenesis of PCOS, providing a new target for treatment [33]. This study confirms that BRCA1 mitigates PCOS through regulating ERS by reducing inflammation and oxidative stress and inhibiting cell apoptosis. However, this study has some limitations. The effect of BRCA1 on PCOS mice was only judged using the sex hormone levels and ovarian tissue morphology of the mice. The effects of BRCA1 on the estrous cycle, body weight, lipid metabolism levels, and insulin resistance must be comprehensively evaluated. The mechanism through which BRCA1 regulates ERS in PCOS needs further systematic research. This study provides a new target, BRCA1, for treating PCOS and suggests the mechanisms of action of BRCA1 in PCOS.

## Conclusions

In summary, this study confirmed that BRCA1 expression level is low in patients with PCOS. BRCA1 overexpression increases sex hormone levels and restores normal ovary morphology in DHEA-induced PCOS mice as well as reduces inflammation, oxidative stress, cell apoptosis, and ERS in PCOS mice and KGN cell models. Treatment with the ERS-inducer TG aggravated inflammation, oxidative stress, and apoptosis in a KGN cell model. These findings demonstrate that BRCA1 alleviates PCOS via inhibiting ERS, reducing inflammation and oxidative stress, and inhibiting apoptosis.

## Supplementary Information

Supplemaentary Material 1. Fig. S1. Phenotypic assessment of Control, PCOS, and PCOS + Lv-BRCA1 mice. **A**. Body-weight changes; **B**. Estrous-cycle stages; **C**. Indices of glucose tolerance and lipid metabolism. Data are shown as mean ± SD. **P < 0.01 ***P < 0.001 vs Control group; #*P* < 0.05, ##*P* < 0.01 vs PCOS group

## Data Availability

The datasets generated during and/or analyzed during the current study are available from the corresponding author upon reasonable request.
