# Supplementary figures and images for: BRCA1 alleviates inflammation, oxidative stress, and ovarian granulosa cell apoptosis by inhibiting endoplasmic reticulum stress, thereby ameliorating polycystic ovary syndrome
Source: Eur J Med Res. 2026 Jan 3;31:186. doi: 10.1186/s40001-025-03734-6 (PMC12866014; doi:10.1186/s40001-025-03734-6)

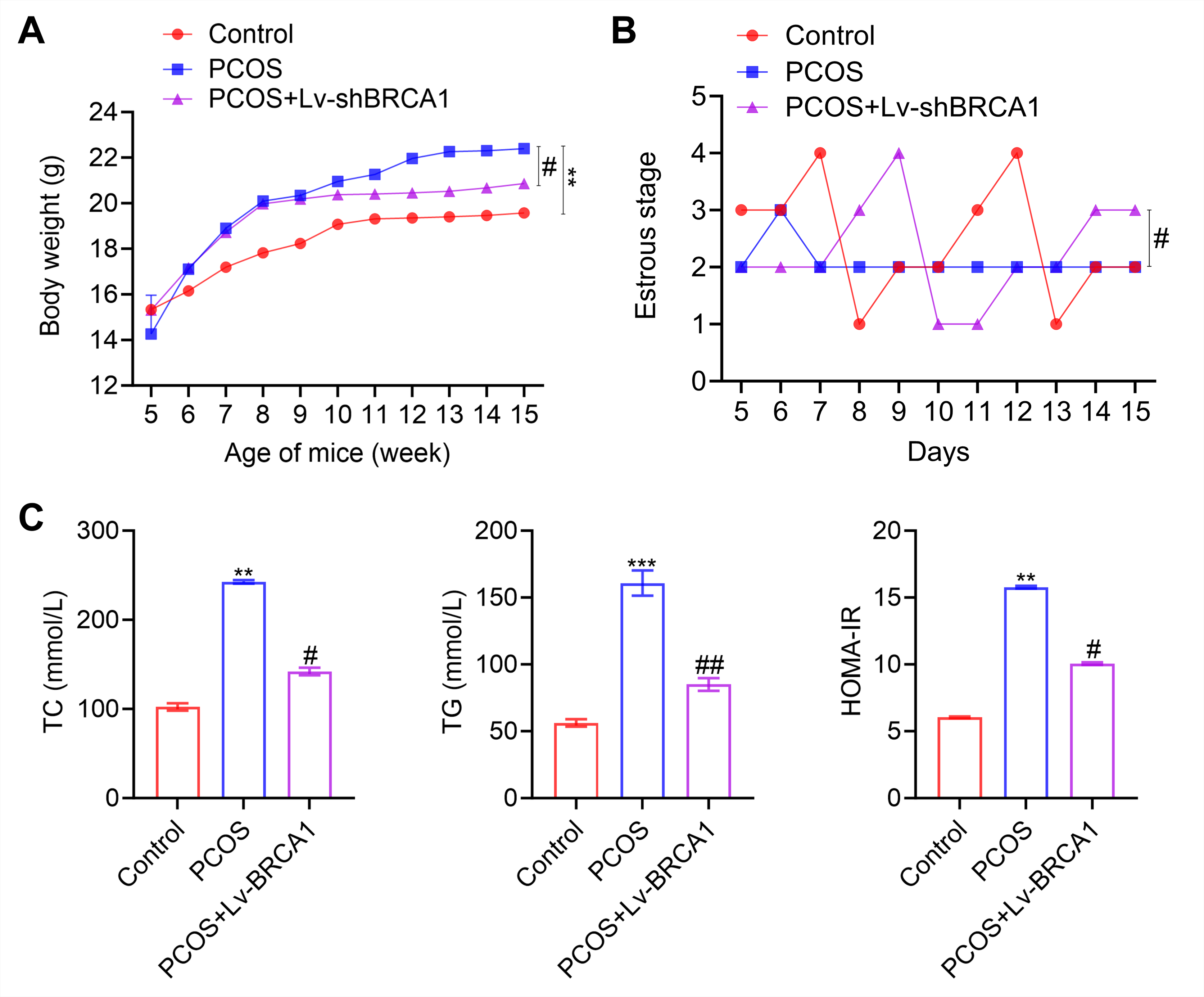

Supplement: Supplementary file 1 — Supplemaentary Material 1. Fig. S1. Phenotypic assessment of Control, PCOS, and PCOS + Lv-BRCA1 mice. A. Body-weight changes; B. Estrous-cycle stages; C. Indices of glucose tolerance and lipid metabolism. Data are shown as mean ± SD. **P < 0.01 ***P < 0.001 vs Control group; #P < 0.05, ##P < 0.01 vs PCOS group [file 40001_2025_3734_MOESM1_ESM.tif]
